# Quantitative Magnetic Resonance Imaging Analysis of Early Markers of Upper Cervical Cord Atrophy in Multiple Sclerosis and Neuromyelitis Optica Spectrum Disorder

**DOI:** 10.1155/2021/9917582

**Published:** 2021-07-09

**Authors:** Iman Adibi, Afshin Najafi, Fouad Merajifar, Neda Ramezani, Hosein Nouri, Nassim Jalilvand, Fereshteh Ashtari, Alireza Vard, Vahid Shaygannejad

**Affiliations:** ^1^Isfahan Neurosciences Research Center, Isfahan University of Medical Sciences, Isfahan, Iran; ^2^Department of Neurology, School of Medicine, Isfahan University of Medical Sciences, Isfahan, Iran; ^3^Network of Immunity in Infection, Malignancy, and Autoimmunity (NIIMA), Universal Scientific Education and Research Network (USERN), Isfahan, Iran; ^4^Department of Bioelectrics and Biomedical Engineering, School of Advanced Technologies in Medicine, Isfahan University of Medical Sciences, Isfahan, Iran; ^5^Medical Image and Signal Processing Research Center, Isfahan University of Medical Sciences, Isfahan, Iran

## Abstract

**Purpose:**

To quantitatively analyze the C2/C3 segments of the spinal cord on magnetic resonance imaging (MRI) scans of neuromyelitis optica spectrum disorder (NMOSD) and relapsing-remitting multiple sclerosis (RRMS) patients in their first five years of the disease and to investigate the intergroup differences regarding markers of spinal cord atrophy and their correlations with expanded disability status scale (EDSS).

**Materials and Methods:**

Twenty NMOSD patients and twenty RRMS patients, within their first five years of the disease, were enrolled in this cross-sectional study. All patients underwent spinal cord MR imaging using 1.5 Tesla systems, and C2/C3 portions of the spinal cord were segmented in the obtained scans. C2/C3 anteroposterior diameter (C2/C3 SC-APD), transversal diameter (C2/C3 SC-TD), and cross-sectional area (C2/C3 SC-CSA) were quantitatively measured using Spinal Cord Toolbox v.4.3.

**Results:**

Three NMOSD patients were seropositive for anti-AQP4 IgG. The mean C2/C3 SC-CSA in NMOSD patients was significantly lower than in RRMS patients. NMOSD patients had significantly lower C2/C3 SC-TDs than RRMS patients. With the three anti-AQP4+ patients excluded from the analysis, C2/C3 SC-TD was negatively correlated with EDSS.

**Conclusion:**

In the early stages of the disease, quantitative evaluation of C2/C3 spinal cord parameters, including cross-sectional area and transversal diameter in NMOSD patients, appears to be of potential diagnostic and prognostic value.

## 1. Introduction

Neuromyelitis optica spectrum disorder (NMOSD) and multiple sclerosis (MS) are chronic autoimmune-mediated diseases of the central nervous system (CNS), characterized by neuroinflammation and neurodegeneration [1, 2]. NMOSD and MS have distinct underlying inflammatory and degenerative processes and have different clinical courses, prognoses, and treatments [3–5]. Spinal cord lesions (SCLs) are common in magnetic resonance imaging (MRI) scans of MS and NMOSD patients. NMOSD SCLs predominantly affect the central gray matter of the spinal cord, where higher levels of aquaporin-4 (AQP4; the immunological target of NMOSD-associated IgG autoantibody) are expressed and often extend over three or more contiguous vertebral bodies in length (i.e., longitudinally extensive transverse myelitis (LETM)) [1, 6].

Spinal cord atrophy (SCA), a hallmark of neurodegeneration, may occur in patients with NMOSD or MS and correlates with progression and worsening of clinical disability [7–9]. SCA may not always be accompanied by spinal cord lesions in MS and NMOSD patients; it also may occur independently of brain pathology [10]. In NMOSD patients, unlike those with RRMS, SCA may be more pronounced than brain atrophy [11, 12]. Although less marked than in NMOSD patients, SCA can indeed be detected in MS patients as early as in the clinically isolated syndrome (CIS) stage [13]. When monitored for five years, the annual rate of SCA was associated with the risk of developing clinically definite MS [14]. The annual rate of SCA in RRMS patients is estimated at around -0.38% and is thought to be lower than that in progressive MS patients [15]. A longitudinal study has shown significant decreases in the mean upper cervical cord cross-sectional area (MUCCA) in NMOSD patients, but not in MS patients, whose atrophic patterns appeared to involve the brain and thalami more prominently [10]. Regarding the disability progression, yearly decreases of the MUCCA in NMOSD patients have shown significant predictive value in that study [11], which has not been confirmed by another study, where authors argue that thoracic (T8/T9 and T9/T10), but not cervical, cross-sectional area is negatively associated with EDSS [16]. They also discuss that EDSS scores of MS patients are negatively associated with cervical spinal cord (C2/C3 and C3/C4) cross-sectional area, but not with thoracic spinal cord cross-sectional area [16]. Interstudy heterogeneities in the disease durations of enrolled patients could have partly contributed to these disputing findings.

MS and NMOSD have different relapse and progression patterns; as such, different patterns of early neurodegeneration in these patients may be reflected through intergroup differences in the spinal cross-sectional area and atrophy volumes. Therefore, quantitative analyses and intergroup comparisons of these parameters may present valuable information concerning the early degenerative events that are subtle enough to miss during regular MRI evaluations. Furthermore, the possible association of these early manifesting parameters with disability progression in MS and NMOSD can offer some prognostic value. The present study is aimed at comparing NMOSD and MS patients within their first five years of the disease in terms of quantitative MRI parameters (i.e., cross-sectional area and transversal and anteroposterior diameters) of the upper cervical spinal cord (at the level of C2/C3) and at determining if any of these parameters are correlated with existing clinical disability.

## 2. Materials and Methods

### 2.1. Participants and Study Setting

In this cross-sectional study, twenty NMOSD patients who met the 2015 criteria for diagnosing NMOSD, proposed by Wingerchuk et al. [17], were enrolled. Twenty patients with definite diagnoses of RRMS, according to the 2017 revisions of the McDonald criteria [18], were also recruited. All participants were chosen from the consecutive patients referred to the MS Clinic of Kashani Hospital, Isfahan, Iran. Included participants for both NMOSD and RRMS groups were patients with (i) definite diagnoses of RRMS or NMOSD, according to the respective diagnostic criteria [17, 18]; (ii) less than five years of disease duration (to minimize the confounding effect of longer disease durations on cervical spinal cord volumes); (iii) no present or previous spine disorders, including arthritis, degenerative disc disease, disc herniation, spinal stenosis, and spondylosis; (iv) no present or previous neurological disorders of the CNS, other than NMOSD and MS; (v) no previous history of radiotherapy; and (vi) no previous history of alcohol abuse. The protocol of this study was approved by the ethical board of Isfahan University of Medical Sciences, and signed informed consent letters were collected.

### 2.2. Clinical and Serological Measures

Demographic information of all patients was documented. Additionally, concerning the confounding effect of variations in patients' body masses, body mass index (BMI) scores for all participants were measured to control this factor upon statistical analyses. Furthermore, all patients underwent routine neurological examinations, and measures of clinical disability (described via expanded disability status scale (EDSS)), duration of the disease, and utilized treatments for each patient were recorded.

Serum samples of NMOSD patients were collected and tested using cell-based assays on slides of fixed cells for anti-AQP4-IgG before the study initiation, and the respective serological data were retrieved from their documents.

### 2.3. Image Acquisitions and Analysis

All patients underwent spinal cord MR imaging using a 1.5 Tesla MR system (Avanto, Siemens, Erlangen, Germany) and an eight-channel phased-array head matrix coil joined to a neck matrix coil to improve the signal-to-noise ratio (SNR). The standard MRI protocols were performed according to the Consortium of MS Centers MRI protocol 2018 revised guidelines [19]. The sagittal T1/T2 (TR = 2500, TE = 82.0, thickness = 3.0 mm, sp = 0, FOV = 230∗230) of the cervical spinal cord and axial T1/T2 (TR = 526.0, TE = 18.0, thickness = 4.0 mm, sp = 0, FOV = 200∗200) through lesions were acquired.

The images were obtained in Digital Imaging and Communications in Medicine (DICOM) format. All the images were converted to the Neuroimaging Informatics Technology Initiative (NIFTI) format to use in the software. Spinal Cord Toolbox v.4.3 was used to quantify the diameters and the mean area of the spinal cord in C2/C3 segments [20]. Primarily, the spinal cord was segmented with propagated cord segmentation settings using T1W images. The segmented image with the original image was used to label the discs and vertebrae to segment C2/C3 automatically. Then, the spinal cord was straightened, registered, and warped into metric objects. All steps were controlled through the FSLeyes viewer, edited in case of any discrepancy and error, and checked by two operators, both blinded to subjects' disease. The area and diameters were reported in square millimeter and millimeter, respectively.

### 2.4. Statistical Analysis

Statistical analyses were performed using the SPSS v.26.0 software (IBM) for Windows. Considering the number of samples in each group (<30), the Mann-Whitney *U* test was utilized to evaluate intergroup differences. Spearman's correlation coefficient was applied when investigating any potential correlation between different spinal cord measures and EDSS. A significance cutoff of 0.05 was set for *p* values.

## 3. Results

### 3.1. Demographic Features

The total number of participants was forty, with twenty in the RRMS group and twenty in the NMOSD group. The mean ages (±SD) and female to male ratios of the two groups were not significantly different. Demographic features of the patients are summarized in Table 1.

### 3.2. Clinical Features

Patients in the RRMS group had longer disease durations than NMOSD patients (3.80 ± 1.36 in the RRMS group vs. 2.10 ± 1.57 in the NMOSD group, *p* = 0.001). Disease durations were not correlated with the EDSS scores of the patients (*p* = 0.454; *r*_s_ = 0.019). No significant difference was noted between the two groups regarding BMI scores. Detailed information on patients' treatment regimens is presented in Table 2. Three patients in the NMOSD group were seropositive for anti-AQP4 IgG.

### 3.3. Quantitative Analyses of the Cervical Spinal Cord Measures

All spinal cord measurements in this study were performed on the C2/C3 spinal cord. Findings from these quantitative analyses are described below.

#### 3.3.1. C2/C3 Spinal Cord Cross-Sectional Area (C2/C3 SC-CSA)

The mean C2/C3 SC-CSA in RRMS patients was significantly higher than that in NMOSD patients (68.33 ± 9.63 mm^2^ vs. 61.86 ± 9.19 mm^2^, *p* value = 0.036) (Table 1); it was also confirmed when the three anti-AQP4+ patients were removed from the analysis (*p* = 0.031). We found no correlation between the C2/C3 SC-CSA and EDSS in either group of the patients (*p* = 0.507, *r*_s_ = −0.157 in the RRMS group and *p* = 0.388, *r*_s_ = −0.204 in the NMOSD group). Similarly, no such correlation was found in the entire study population, regardless of their disease types (*p* = 0.198, *r*_s_ = −0.208). C2/C3 SC-CSA in the whole study population showed a weak positive correlation with disease duration (*p* = 0.043; *r*_s_ = 0.350) that did not survive the removal of anti-AQP4+ patients from the analysis (*p* = 0.133, *r*_s_ = 0.252); further, it was diminished when inspected in each of the groups distinctively (*p* = 0.677, *r*_s_ = 0.106 in the RRMS group and *p* = 0.102, *r*_s_ = 0.423 in the NMOSD group).

#### 3.3.2. C2/C3 Spinal Cord Transversal and Anteroposterior Diameters

The mean C2/C3 SC-TD was lower in NMOSD patients than in RRMS patients (11.16 ± 1.10 mm vs. 12.03 ± 0.84 mm; *p* = 0.004) (Table 1); this was also confirmed when anti-AQP4+ patients were omitted from the analysis (*p* = 0.005). However, we did not observe any difference between the two groups regarding C2/C3 SC-APD, 7.54 ± 0.89 mm in the RRMS group and 7.44 ± 0.84 mm in the NMOSD group (*p* = 0.626).

Although nonsignificant, there was an indication of a weak correlation between the C2/C3 SC-TD and EDSS when analyzing the entire population of the study (*p* = 0.081; *r*_s_ = −0.279); this negative correlation turned statistically significant when anti-AQP4+ patients were excluded from the analysis (*p* = 0.027; *r*_s_ = −0.365). C2/C3 SC-APD did not yield any correlation with EDSS scores of the patients (*p* = 0.604; *r*_s_ = −0.084). On this matter, when data from each group were analyzed separately, none of these diametric measures of the spinal cord were correlated with EDSS (*p* = 0.311, *r*_s_ = −0.238 and *p* = 0.789, *r*_s_ = −0.064 for C2/C3 SC-TD in RRMS and NMOSD patients, respectively, and *p* = 0.858, *r*_s_ = −0.043 and *p* = 0.287, *r*_s_ = −0.244 for C2/C3 SC-APD in RRMS and NMOSD patients, respectively). Neither were they correlated with disease duration, whether when analyzed in the total population of the study or patients from each of the disease groups, separately (*p* = 0.671, *r*_s_ = −0.108, *p* = 0.126, *r*_s_ = 0.398, and *p* = 0.090, *r*_s_ = 0.295 for C2/C3 SC-TDs in the RRMS group, NMOSD group, and all patients, respectively, and *p* = 0.489, *r*_s_ = 0.174; *p* = 0.977, *r*_s_ = −0.008; and *p* = 0.483, *r*_s_ = 0.124 for C2/C3 SC-APDs in the RRMS group, NMOSD group, and all patients, respectively).

## 4. Discussion

This study showed that in patients with a disease duration of less than five years, the UCCA was significantly lower in NMOSD than in RRMS. Furthermore, in this study, the mean C2/C3 SC-TD was significantly lower in NMOSD patients than in RRMS patients. Lower C2/C3-SC-TDs may be correlated with higher EDSS scores at the time of measurement.

### 4.1. Spinal Cord Atrophy in MS

Spinal cord atrophy is an important radiological feature in MS, particularly in progressive phases of the disease [21], indicating an extensive axonal loss in the disease course after the initially more prominent demyelination [22, 23]. Longitudinal studies have shown that spinal cord atrophy in RRMS patients is an independent predictor of disease progression and clinical disability, regardless of the reduction in the whole brain volume or lesion load [24, 25]. However, spinal cord atrophy is not limited to patients with progressive MS. Reduction in UCCA is also present in patients with early MS and CIS patients [13]. It is not thoroughly clarified if the magnitude of association between spinal cord atrophy and the disability of patients alters during the disease course. Future studies should compare the value of spinal cord atrophy in predicting disability between patients with RRMS and progressive MS. Given that spinal cord atrophy in MS patients can progress several years earlier than both brain volume loss [8, 21] and disability progression [26, 27], early spinal cord atrophy can serve as a marker of ongoing subclinical progression and long-term clinical progression.

### 4.2. Spinal Cord Atrophy in NMOSD

Upper cervical spinal cord lesions and atrophy are common in NMOSD patients' MRI scans and are associated with clinical disability and sensory and motor dysfunctions, as well as EDSS [7]. In these patients, reduced volume and cross-sectional area (atrophy) of the upper cervical cord may occur independently of spinal cord lesions or relapses and are notably correlated with clinical disability [28]. This subtle progression of spinal cord atrophy is not detectable in routine, conventional MRI evaluations and necessitates further investigations applying quantitative methods to monitor less apparent spinal cord volume changes.

### 4.3. Selection of C2/C3 Spinal Cord

In the present study, we incorporated quantitative analyses of the cervical spinal cord at the level of C2/C3. According to a recent study, significant tissue loss and atrophic changes in two different levels, i.e., C2-C4 segments and T1-T3 segments, are significantly associated with clinical disability in NMOSD patients [7]. Furthermore, a large-scale study on primary progressive MS (PPMS) patients showed that measures obtained at different spinal cord segments had comparable clinical correlates. Incorporating brain MRI scans to acquire upper cervical measures appeared more effortless and accurate than utilizing spinal MRI scans, given the lower variability of the surrounding cerebrospinal fluid (CSF) signals in the former [29]. On this matter, when choosing the most reliable level of the spinal cord to measure, the width of CSF at that level (to maximize the CSF/spinal cord contrast), variations in the cross-sectional area of the spinal cord (to ensure the reliability of the measurements), and prevalence of disc protrusions are important. Hence, the C2-C3 segment is an appropriate level of interest in UCCA measurements in quantitative analysis of spinal cord atrophy [30].

### 4.4. Disease Duration and Spinal Cord Measures of Atrophy

Duration of the disease may indirectly affect the cervical spinal cord measures in patients with RRMS or NMOSD. Thus, by utilizing the appropriate selection criterion to include only patients within their first five years of the disease, we attempted to limit the confounding effect of the disease duration on the spinal cord measures as much as possible. This maneuver also enabled us to refer the obtained results in this study to the early stages of NMOSD and RRMS. Therefore, although the observed difference between the two groups in terms of disease duration was statistically significant, it was not clinically meaningful, as all patients were in the first five years of their disease. Despite having a shorter mean disease duration, NMOSD patients had higher EDSS scores than RRMS patients.

### 4.5. C2/C3 SC-CSA in the Early Stages of the Disease

In a longitudinal study by Liu et al., changes in MUCCA over a one-year follow-up period reflected the progression rate in NMOSD patients but not in MS patients. Baseline MUCCA values in both groups were significantly different from those observed in healthy controls, while no differences were detected between the MS and NMOSD patients [31]. However, our study showed significantly lower C2/C3 SC-CSAs in NMOSD patients than RRMS patients in the early stages of these disorders. Shorter disease duration of patients enrolled in our study (i.e., less than five years), using different anatomical indices (C2/C3 spinal cord in our study and MUCCA in the study by Liu et al.), and applying different softwares for quantitative analysis utilized may partly explain these differences. Nevertheless, our findings suggested that changes in the upper cervical cord cross-sectional area could be prominent early findings (in the first five years) in NMOSD patients.

A recent study by Nakamura et al. showed that decreased C2/C3 SC-CSA was correlated with higher EDSS scores in MS patients, who had a mean disease duration of more than 14 years (including RRMS and PPMS patients). They could not show this correlation in patients with NMOSD [16]. It seems that disease duration and course are important factors in applying UCCA as a marker of progression and disability in MS patients, as in our patients with a mean disease duration of less than four years, C2/C3 cross-sectional area was not correlated with EDSS.

### 4.6. C2/C3 SC-TD in the Early Stages of the Disease

Although NMOSD patients had a mean disease duration of approximately 1.5 years less than RRMS patients, they had significantly lower C2/C3 SC-TD values than RRMS patients (12.03 ± 0.84 mm vs. 11.16 ± 1.10 mm, *p* = 0.004). C2/C3 SC-APD differences were not significant between RRMS and NMOSD patients. This difference may imply the affected tracts of spinal cord white matter, which are evaluated in measuring C2/C3 SC-TD, including lateral spinothalamic tracts and lateral corticospinal tracts, in which axonal degeneration and atrophy seem to be present from early phases of NMOSD [1].

### 4.7. Limitations

This study is a preliminary cross-sectional study with relatively small sample size, limiting our power of subgroup analyses (e.g., comparing the obtained variables between anti-AQP4-IgG-seropositive and seronegative NMOSD patients). Therefore, further studies with larger sample sizes are warranted to validate our results and perform more detailed statistical analyses that allow for identifying different progression phenotypes (e.g., the study by Moccia et al. [32]). Further, given that about 7-42% of seronegative NMOSD patients are positive for anti-MOG IgG [33], another limitation is that anti-MOG IgG was not checked in our seronegative NMOSD patients.

## 5. Conclusion

Atrophic changes involving the upper cervical cord in the early stages of the disease (i.e., the first five years) are more prominent in NMOSD patients than in RRMS patients. Quantitative analysis of C2/C3 spinal cord measures, such as C2/C3 SC-TD, may unravel pathological alterations in NMOSD patients, indicative of atrophy, and may be correlated with EDSS. Therefore, further assessments of the applicability of these quantitative measures on MRI scans of patients appear necessary in future studies as these markers seem to be of considerable diagnostic and prognostic value in the early stages of the disease.

## Figures and Tables

**Table 1:** Demographic, clinical, and quantitative MRI measures of the participants.

	RRMS	NMOSD	*p* value
Female/male (%)	14 (70)/6 (30)	15 (75)/5 (25)	0.723
Age (mean ± SD) (years)	37.80 ± 9.44	35.35 ± 13.96	0.52
Disease duration (years)	3.80 ± 1.36	2.10 ± 1.57	0.001^∗^
EDSS	2.35 ± 1.50	3.45 ± 1.73	0.046^∗^
BMI	25.51 ± 4.91	25.10 ± 4.97	0.813
C2/C3 SC-CSA	68.33 ± 9.63 mm^2^	61.86 ± 9.19 mm^2^	0.036^∗^
C2/C3 SC-TD	12.03 ± 0.84 mm	11.16 ± 1.10 mm	0.004^∗^
C2/C3 SC-APD	7.54 ± 0.89 mm	7.44 ± 0.84 mm	0.626

^∗^Statistically significant difference. BMI: body mass index; C2/C3 SC-APD: C2/C3 spinal cord anteroposterior diameter; C2/C3 SC-CSA: C2/C3 spinal cord cross-sectional area; C2/C3 SC-TD: C2/C3 spinal cord transversal diameter; EDSS: expanded disability status scale.

**Table 2:** Treatment-associated data of patients.

Treatment	RRMS patients (%)	NMOSD patients (%)
Interferon-*β* 1a	9 (45)	0 (0)
Fingolimod	2 (10)	0 (0)
Rituximab	5 (25)	13 (65)
Dimethyl fumarate	3 (15)	0 (0)
Teriflunomide	1 (5)	0 (0)
Azathioprine	0 (0)	7 (35)

## Data Availability

The data that support the findings of this study are available from the corresponding author, Vahid Shaygannejad, upon reasonable request.

## References

[1] Wingerchuk D. M., Lennon V. A., Lucchinetti C. F., Pittock S. J., Weinshenker B. G. (2007). The spectrum of neuromyelitis optica. *The Lancet Neurology*.

[2] Polman C. H., Reingold S. C., Banwell B. (2011). Diagnostic criteria for multiple sclerosis: 2010 revisions to the McDonald criteria. *Annals of Neurology*.

[3] Kawachi I., Lassmann H. (2017). Neurodegeneration in multiple sclerosis and neuromyelitis optica. *Journal of Neurology, Neurosurgery & Psychiatry*.

[4] Palace J., Leite M. I., Nairne A., Vincent A. (2010). Interferon beta treatment in neuromyelitis optica: increase in relapses and aquaporin 4 antibody titers. *Archives of Neurology*.

[5] Min J. H., Kim B. J., Lee K. H. (2012). Development of extensive brain lesions following fingolimod (FTY720) treatment in a patient with neuromyelitis optica spectrum disorder. *Multiple Sclerosis Journal*.

[6] Kim H. J., Paul F., Lana-Peixoto M. A. (2015). MRI characteristics of neuromyelitis optica spectrum disorder: an international update. *Neurology*.

[7] Cacciaguerra L., Valsasina P., Mesaros S. (2020). Spinal cord atrophy in neuromyelitis optica spectrum disorders is spatially related to cord lesions and disability. *Radiology*.

[8] Lukas C., Knol D. L., Sombekke M. H. (2015). Cervical spinal cord volume loss is related to clinical disability progression in multiple sclerosis. *Journal of Neurology, Neurosurgery & Psychiatry*.

[9] Schlaeger R., Papinutto N., Panara V. (2014). Spinal cord gray matter atrophy correlates with multiple sclerosis disability. *Annals of Neurology*.

[10] Moccia M., Ruggieri S., Ianniello A., Toosy A., Pozzilli C., Ciccarelli O. (2019). Advances in spinal cord imaging in multiple sclerosis. *Therapeutic Advances in Neurological Disorders*.

[11] Liu Y., Duan Y., Huang J. (2018). Different patterns of longitudinal brain and spinal cord changes and their associations with disability progression in NMO and MS. *European Radiology*.

[12] Ciccarelli O., Cohen J. A., Reingold S. C. (2019). Spinal cord involvement in multiple sclerosis and neuromyelitis optica spectrum disorders. *The Lancet Neurology*.

[13] Biberacher V., Boucard C. C., Schmidt P. (2015). Atrophy and structural variability of the upper cervical cord in early multiple sclerosis. *Multiple Sclerosis Journal*.

[14] Brownlee W. J., Altmann D. R., Alves da Mota P. (2017). Association of asymptomatic spinal cord lesions and atrophy with disability 5 years after a clinically isolated syndrome. *Multiple Sclerosis Journal*.

[15] Tsagkas C., Magon S., Gaetano L. (2018). Spinal cord volume loss: a marker of disease progression in multiple sclerosis. *Neurology*.

[16] Nakamura Y., Liu Z., Fukumoto S. (2020). Spinal cord involvement by atrophy and associations with disability are different between multiple sclerosis and neuromyelitis optica spectrum disorder. *European Journal of Neurology*.

[17] Wingerchuk D. M., Banwell B., Bennett J. L. (2015). International consensus diagnostic criteria for neuromyelitis optica spectrum disorders. *Neurology*.

[18] Thompson A. J., Banwell B. L., Barkhof F. (2018). Diagnosis of multiple sclerosis: 2017 revisions of the McDonald criteria. *The Lancet Neurology*.

[19] The Consortium of Multiple Sclerosis Centers MRI Task Force Consortium of MS Centers (2018). MRI protocol and clinical guidelines for the diagnosis and follow-up of MS: 2018 revised guidelines. https://cdn.ymaws.com/mscare.site-ym.com/resource/collection/9C5F19B9-3489-48B0-A54B-623A1ECEE07B/2018MRIGuidelines_booklet_with_final_changes_0522.pdf.

[20] de Leener B., Lévy S., Dupont S. M. (2017). SCT: spinal cord toolbox, an open-source software for processing spinal cord MRI data. *NeuroImage*.

[21] Kearney H., Miller D. H., Ciccarelli O. (2015). Spinal cord MRI in multiple sclerosis--diagnostic, prognostic and clinical value. *Nature Reviews Neurology*.

[22] Bot J. C., Blezer E. L., Kamphorst W. (2004). The spinal cord in multiple sclerosis: relationship of high-spatial-resolution quantitative MR imaging findings to histopathologic results. *Radiology*.

[23] Ganter P., Prince C., Esiri M. M. (1999). Spinal cord axonal loss in multiple sclerosis: a post-mortem study. *Neuropathology and Applied Neurobiology*.

[24] Daams M., Weiler F., Steenwijk M. D. (2014). Mean upper cervical cord area (MUCCA) measurement in long-standing multiple sclerosis: relation to brain findings and clinical disability. *Multiple Sclerosis Journal*.

[25] Kearney H., Rocca M. A., Valsasina P. (2014). Magnetic resonance imaging correlates of physical disability in relapse onset multiple sclerosis of long disease duration. *Multiple Sclerosis Journal*.

[26] Rocca M. A., Sormani M. P., Rovaris M. (2017). Long-term disability progression in primary progressive multiple sclerosis: a 15-year study. *Brain*.

[27] Tsagkas C., Magon S., Gaetano L. (2019). Preferential spinal cord volume loss in primary progressive multiple sclerosis. *Multiple Sclerosis Journal*.

[28] Ventura R. E., Kister I., Chung S., Babb J. S., Shepherd T. M. (2016). Cervical spinal cord atrophy in NMOSD without a history of myelitis or MRI-visible lesions. *Neurology-Neuroimmunology Neuroinflammation*.

[29] Moccia M., Valsecchi N., Ciccarelli O., Van Schijndel R., Barkhof F., Prados F. (2020). Spinal cord atrophy in a primary progressive multiple sclerosis trial: improved sample size using GBSI. *NeuroImage: Clinical*.

[30] Losseff N. A., Webb S. L., O'Riordan J. I. (1996). Spinal cord atrophy and disability in multiple sclerosis. *Brain*.

[31] Liu Y., Wang J., Daams M. (2015). Differential patterns of spinal cord and brain atrophy in NMO and MS. *Neurology*.

[32] Moccia M., Prados F., Filippi M. (2019). Longitudinal spinal cord atrophy in multiple sclerosis using the generalized boundary shift integral. *Annals of Neurology*.

[33] Hor J. Y., Asgari N., Nakashima I. (2020). Epidemiology of neuromyelitis optica spectrum disorder and its prevalence and incidence worldwide. *Frontiers in Neurology*.

